# KLF4 is an epigenetically modulated, context-dependent tumor suppressor

**DOI:** 10.3389/fcell.2024.1392391

**Published:** 2024-07-29

**Authors:** Raffaele Frazzi

**Affiliations:** Molecular Pathology Laboratory, Azienda Unità Sanitaria Locale–IRCCS di Reggio Emilia, Reggio Emilia, Italy

**Keywords:** KLF4, epigenetic regulation, DNA methylation, histone marks, tumor suppressor

## Abstract

The epigenetic layer of regulation has become increasingly relevant in the research focused on tumor suppressors. *KLF4* is a well-described zinc-finger transcription factor, mainly known for its role in the acquisition of cell pluripotency. Here we report and describe the most relevant epigenetic regulation mechanisms that affect *KLF4* expression in tumors. CpG island methylation emerges as the most common mechanism in several tumors including lung adenocarcinoma, hepatocellular carcinoma, non-Hodgkin lymphomas, among others. Further layers of regulation represented by histone methylation and acetylation and by non-coding RNAs are described. Overall, *KLF4* emerges as a crucial target in the fight against cancer.

## 1 Introduction

**FIGURE 1:**
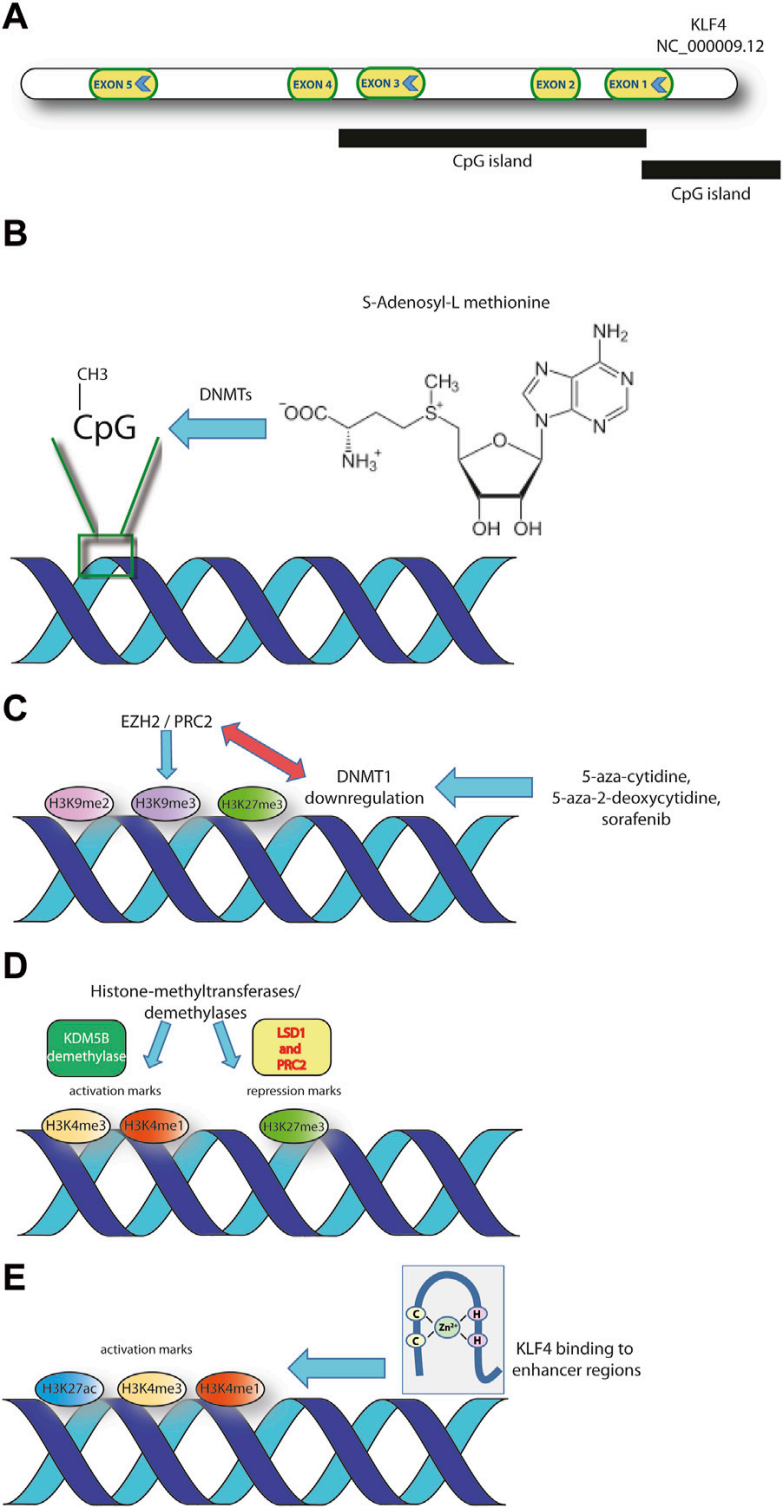
The main layers of *KLF4* epigenetic regulation discussed in the text are represented. **(A)** Structure of *KLF4* gene (GRCh38.p14 assembly). **(B)** Methylation at specific CpG sites within *KLF4* CpG islands. S-adenosyl-methionine is the universal methyl group donor in DNA- and histone-methylation reactions. **(C)** Methylation of specific histone residues represent repressory marks at specific DNA sequences. The interaction of DNMT1 with histone methyltransferases affects the chromatin architecture. **(D)** Histone methylation on specific histone residues within regulatory regions may represent activatory or repressory marks. The process is finely tuned through histone methyltransferases and demethylases. **(E)** KLF4 binding to enhancer regions, enriched in histone activation marks (H3K27ac, H3K4me1, H3K4me3), is a characteristic of the regulatory footprint of some tumors.

Krüppel-like factor 4 (KLF4) belongs to a family of transcription factors (TFs) playing a variety of functions and having a significant potential in cancer development and progression. The gene is located on human chromosome 9 and contains 5 exons (Figure 1A) (NCBI, 2023). KLF4 is a zinc finger-containing transcription factor and is among the four factors able to induce pluripotent stem cells (together with OCT3/4, SOX2 and c-MYC) (Dang et al., 2000; Kalra et al., 2011). The three zinc finger-motifs are adjacent and located at the C-terminus of the sequence. At the N-terminus there is a transactivation domain adjacent to a repression domain. These, together, define the specificity of the transcriptional regulation and the efficiency of DNA binding, in cooperation with other factors (Ghaleb and Yang, 2017). The gene contains also two nuclear localization signals, one close to the most amino-terminal zinc finger domain, while the second spanning the first and half of the second zinc finger domain (Shields and Yang, 1997). The simultaneous presence of activatory and repressory domains within the *KLF4* gene allows this transcription factor to exert its specific activity on different targets, according to the specific tissue distribution (Li et al., 2015). The current knowledge points towards *KLF4* as a tumor suppressor in both solid and hematological tumors, where the gene is silenced by means of a variety of mechanisms including DNA hypermethylation, interactions with miRNAs, histone modifications and others, described subsequently in the manuscript (Guan et al., 2010; Frazzi et al., 2017; Frazzi et al., 2022). A few evidences exist though supporting a role as an oncogene. For instance, the malignant progression of ductal carcinoma *in situ* (DCIS) is driven by the overexpression of *KLF4* (Damaghi et al., 2021). This transcription factor, together with NF-kB activation, plays a role in the initial phase of the development of one of the most diagnosed human cancers: breast carcinoma. Thus, in DCIS, KLF4 plays an oncogenic role. The available literature reports a number of different epigenetic mechanisms that have been involved in the regulation of this zinc-finger transcription factor. In the present manuscript, the main mechanisms of epigenetic modulation of *KLF4* expression in tumor cells are described and commented. Furthermore, some epigenetic modifications linked to the regulatory and topological functions of KLF4 TF are outlined. These histone modifications represent epigenetic marks that directly influence not only *KLF4* expression but also its binding to targets. The literature up to 2023 spans a variety of tumors of different histological origins and unveils recurrent epigenetic layers of modulation.

## 2 Epigenetic regulation of gene expression by means of methylation

DNA methylation can mediate gene inactivation through different mechanisms (Figure 1B). It can attract CpG-methylated-DNA binding proteins or histone deacetylases (HDACs) and it may induce variations in histone methylation (Jin et al., 2011; Patra et al., 2011). The epigenetic layer of regulation has been demonstrated in lymphoma cells, were *KLF4* is consistently hypermethylated in B lymphocytes sorted from diffuse large-B cell lymphomas (DLBCL), follicular lymphomas (FL) and non Hodgkin lymphoma (NHL) cell lines (Guan et al., 2010; Frazzi et al., 2017; Frazzi et al., 2022). The evidence that peripheral blood mononuclear cells from healthy donors display a consistently demethylated *KLF4* promoter suggests that the hypermethylated phenotype is associated to B cell malignancy (Frazzi et al., 2022).

Burkitt lymphoma is another subtype where KLF4 oncosuppressive role has been recently demonstrated (Bialopiotrowicz et al., 2020). Histone H3K27 and DNA methylation decrease in BL cells led to the reactivation of the methylation-regulated tumor suppressor genes: Cyclin Dependent Kinase Inhibitor 2B (*CDK2NB*), *KLF4*, Inhibitor of DNA Binding 4 (*ID4*), and Thioredoxin Interacting Protein (*TXNIP*) (Bialopiotrowicz et al., 2020).

Furthermore, KLF4 is a known partner of YY1 in leukocytes and B-NHL. The evidences emerged especially in the last 7 years, demonstrate a constitutive interaction between KLF4 and YY1. In this context, KLF4 acts as an oncogene in B-NHL [(Khan et al., 2019; Morales-Martinez et al., 2019; Morales-Martinez et al., 2020; Montecillo-Aguado et al., 2021)].

*KLF4* emerges also in a recent bioinformatics analysis of lung adenocarcinoma (including 497 tumors and 54 adjacent normal tissue samples). The low expression of *KLF4* (together with other hub-genes) correlated significantly with the poor overall survival (OS) of lung cancer patients (Cai et al., 2022). *KLF4* resulted downregulated due to increased miRNA and hypermethylation (Cai et al., 2022). These data confirm the previous ones published on NHL pointing towards the epigenetic regulation of *KLF4* gene expression.

Approved for the therapy of renal cell, differentiated thyroid and hepatocellular carcinomas, sorafenib exerts an antiproliferative and antiangiogenic activity through the inhibition of serine/threonine and tyrosine kinases (Gong et al., 2017). Sorafenib acts by modulating epigenetic pathways involving DNA methyltransferase 1 (DNMT1) and KLF4 (Figure 1C). Upon DNMT1 inhibition, the expression of metallothionein 1G (*MT1G*) results upregulated (Wei et al., 2022). MT1G belongs to the family of metallothioneines and inhibits several types of human cancers as thyroid, colorectal, pancreatic and its upregulation is associated to sorafenib treatment (Houessinon et al., 2016; Zeng et al., 2018). The *MT1G* expression increases upon sorafenib and also parallels the dysregulation of two crucial genes: *KLF4* and Carbonic Anhydrase 9 (*CA9*). *KLF4* results upregulated and suppresses *CA9*, through the inhibition of Hypoxia Inducible Factor 1 alfa (*HIF-1α*). KLF4 also downregulates two more targets of the HIF-1α axis (Vascular Endothelial Growth Factor A and Solute Carrier Family 2 Member 1), possibly by reducing HIF-1α protein levels (Wei et al., 2022).

Epigenetic silencing has been also demonstrated in T-cell acute lymphoblastic leukemia (T-ALL), where *KLF4* serves as tumor suppressor in experimental animal models and xenografts generated using lymphoblasts from pediatric T-ALL patients (Shen et al., 2017; Chen et al., 2021).

*KLF4* emerged also in a panel of epigenetically regulated genes in colorectal cancer (CRC) (Li et al., 2021). The differentially methylated regions located within the promoters of *TNNI2*, *PAX8*, *GUF1*, *KLF4*, *EVI2B*, *CEP112* and long non-coding RNA AC011298 genes are associated with the probability of liver metastasis in CRC patients. When analyzed through a model of predictive performance (leave-one-out cross validation; LOOCV), the area under the curve (AUC) was 0.701 while the ability to distinguish metastasis of different sites had an AUC = 0.933 (Li et al., 2021).

Loss of heterozigosity and promoter hypermethylation were demonstrated in pancreatic cancer as the mechanisms responsible for the downregulation of *KLF4* (Xie et al., 2017). Thus, the activity of DNMT1 is specifically linked to *KLF4* downregulation. The immunohistochemical (IHC) staining on a total of 84 human pancreatic samples demonstrates that DNMT1 expression inversely correlates with KLF4 (Xie et al., 2017).

More consistent data on the direct role of DNMT1 in controlling *KLF4* expression were demonstrated in prostate cancer (PCa). The ability of prostate cell to undergo epithelial to mesenchimal transition (EMT) is key in driving malignant transformation (Lee et al., 2016). The induction of EMT initiates a self-renewal mechanism leading to the formation of the cancer stem cells (CSCs) (Mani et al., 2008; Jung et al., 2013). Interestingly, the downregulation of DNMT1 drives EMT and promotes CSCs formation in PCa. The histone demethylation following 5-azacytidine treatment (H3K9me3 and H3K27me3 specifically) of the *KLF4* promoter is one of the downstream effects of DNMT1 reduction (Lee et al., 2016). The effects of DNMT1 downregulation are possibly due to the known function of this enzyme in maintaining the architecture of large heterochromatic regions. This is achieved also by modulating the histone H3 methylation and accompanied by an altered nuclear architecture (Espada et al., 2004). EZH2 (enhancer of zeste homolog 2), a member of the polycomb repressor complex-2 (PRC2) is a histone lysine methyltransferase known to interact directly with DNMT1 (Figure 1C). Moreover, HP1 (heterochromatin protein 1) represents a link between heterochromatin and the methylation machinery, interacting with DNMT1 directly and mediating the silencing of euchromatic genes (Jin et al., 2011). Preliminary evidences also show that *KLF4*, together with *Zeb2* and N-cadherin increased expression are associated to advanced PCa. These last evidences support the tumor promoting role of *KLF4* (Lee et al., 2016).

Preliminary data on 52 human samples show that *KLF4* expression is low in high grade dysplasia and early stage esophageal squamous cell carcinoma (ESCC), while it becomes prevalent in high grade ESCC (Yang and Katz, 2016). However, *KLF4* levels increased with tumor size and with the presence of nodal metastases compared to the ones without metastatic spread. One of the proposed mechanisms for low *KLF4* expression, also in ESCC, is promoter methylation (Yang and Katz, 2016).

Oral squamous cell carcinoma (OSCC) is another cancer where the epigenetic regulation of *KLF4* has been demonstrated. The classical function of tumor suppressor is studied through gene expression and promoter methylation approaches (Li et al., 2015). IHC demonstrates that KLF4 staining decreases going from well-differentiated to moderately differentiated and to poorly differentiated OSCC (n = 39). The quantitative methylation of the two CpG islands localized upstream of the transcription starting site consistently display a decreasing methylation percentage in the order: OSCC > dysplasia and healthy oral mucosa. The overexpression of *KLF4* leads to the inhibition of tumor growth, cell proliferation and angiogenesis *in vitro* and in tumor xenografts, confirming its tumor suppressive role (Li et al., 2015).

The demonstration that *KLF4* transcription is modulated through epigenetic mechanisms has been obtained also in hepatocellular carcinoma (HCC). A non-promoter CpG island located within the *KLF4* sequence (proximal to the retained intron of KLF4-003, a non coding transcript of the *KLF4* gene) has been pyrosequenced (Zhou et al., 2015). KLF4-003 was downregulated in HCC tumor samples compared to their normal counterparts (n = 54) and this was significantly associated with HCC recurrence but no other clinical parameters. The hypermethylation of the proximal non-promoter CpG island is likely the mechanism responsible for the KLF4-003 reduced expression (Zhou et al., 2015). Overall, the reprogramming towards pluripotency is considered a promoter for HCC progression and is mediated by promoter methylation regulation of key TFs (Wang et al., 2013).

*KLF4* is downregulated also in lung cancer compared to nontumor tissues, presenting the characteristics of tumor suppressor. Furthermore, *KLF4* deletion promotes lung tumor formation and progression in mouse models. The downregulation is achieved by means of epigenetic mechanisms and, to a little extent, genetic mutations (Yu et al., 2016). Histone deacetylase (HDAC) inhibition seems to be the most relevant mechanism controlling *KLF4* expression in lung cancer cell lines A549 and H460. The rationale is given by the fact that HDAC1 and HDAC3 may interact with *KLF4* modulating its transcription (Yu et al., 2016).

Head and neck squamous cell carcinoma (HNSCC) radioresistant tumors display a higher methylation compared to non resistant ones (Chen et al., 2015). This methylation phenotype affects the gene expression profile and is confirmed by human HNSCC datasets from the TCGA where an increase in DNA methylation is associated to radiation resistance (Chen et al., 2015).

Promoter hypermethylation is the mechanism responsible for *KLF4* downregulation also in urothelial cancer, renal cell carcinoma, medulloblastoma, colorectal adenoma-carcinoma and gastric cancer (Choi et al., 2006; Cho et al., 2007; Xu et al., 2008; Nakahara et al., 2010; Li et al., 2013; Li et al., 2014), making the control of gene expression by means of promoter methylation a general strategy adopted by different tumors.

Interestingly, NOTCH1 can repress *KLF4* expression through the formation of a repressory complex on the gene. This complex includes LSD1 histone demethylase and PRC2, a methyl transferase responsible for methylation of H3K27 histone. The deposition of the repressory mark H3K27me3 can explain the repressory function exerted by the NOTCH1-containing PRC2 complex on *KLF4* gene (Han et al., 2017). The repression of *KLF4* transcription may also be exerted through the epigenetic silencing due to H3K4 demethylation by KDM5B in acute myeloid leukemia (Faber et al., 2013). Collectively, these evidences demonstrate how the chromatin conformational changes mediated by histone methylation/demethylation represent a mechanism adopted by cancer cells to downregulate *KLF4* (Figure 1D).

## 3 Epigenetic regulation of gene expression by means of miRNAs and circular RNA

**FIGURE 2:**
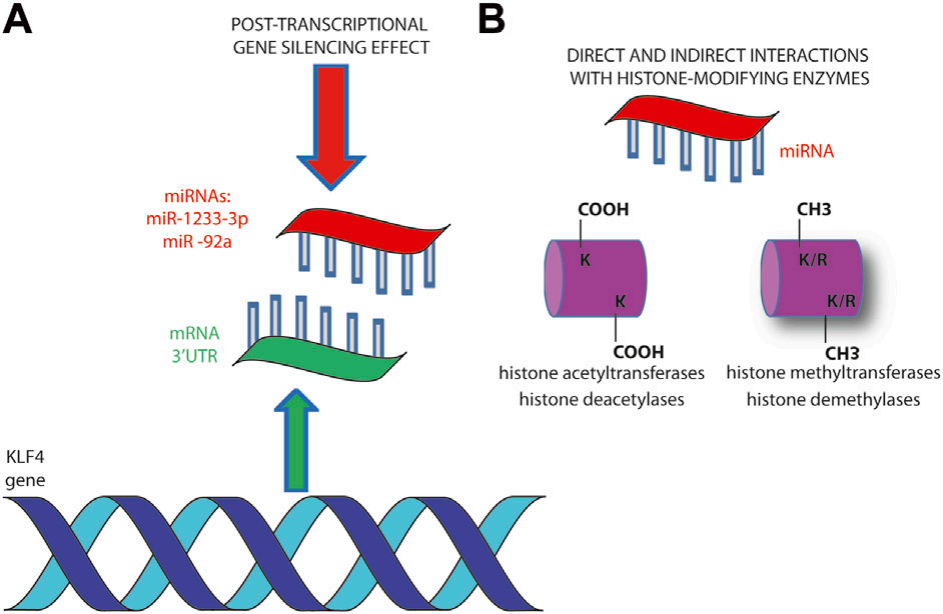
miRNAs regulate *KLF4* gene expression at various levels. **(A)** Post-transcriptional hybridization to 3’-UTR has been demonstrated to be the main mechanism of miR-1233-3p and miR -92a in downregulating *KLF4* expression **(B)**. The interaction of miRNAs with histone modifying enzymes (writers and erasers) regulates chromosomes architecture and affects gene expression. It can be direct through the interaction with histone proteins or by affecting the expression of histone modifying enzymes (Szczepanek and Tretyn, 2023).

The gene regulation by miRNAs is a consolidated mechanism available for epigenetic modulation of gene expression in cancer cells (Ebrahimi et al., 2023; Szczepanek and Tretyn, 2023). Among the several examples of transcriptome programs affected by miRNAs, here are reported some examples that specifically involve *KLF4*. The miR-302/367 cluster is responsible for the reprogramming of glioblastoma U87 cells towards a more benign phenotype (Yang et al., 2015). The epigenetic reprogramming potential of miR-302/367 cluster can be applied to shift cancer cells abolishing their tumorigenic characteristics through a variety of targets. The pluripotency transcription factors OCT3/4, SOX2, KLF4, and c-MYC (OSKM) result downregulated upon miRNA cluster induction and, together with POU3F2, SALL2 and OLIG2, are required for the maintenance of glioblastoma stem-like tumor propagating cells (Yang et al., 2015) (Figure 2).

The same is true in CRC, where a reprogramming mediated by exogenous miR-302s and miR-369s leads to the expression of pluripotency factors. The re-expression of OSKM may lead to reprogramming of differentiated somatic cells back to pluripotent stem cells (Miyoshi et al., 2010; Ogawa et al., 2015). The suppression of the malignant phenotype that follows is achieved through promoter demethylation of oncosuppressor *p16INK4A* pathway and epigenetic alterations of the PRC2 affecting H3K27 methylation (Miyoshi et al., 2010) (Figure 2).

The epigenetic regulation mediated by miRNAs has been reported also in liver cancer, lung adenocarcinoma, breast cancer, CRC, gastric cancer, glioblastoma (Ogawa et al., 2015; Yang et al., 2015; Vidal et al., 2016; Chen et al., 2018; Lu et al., 2020; Cai et al., 2022; Jiang et al., 2023).

Circular RNA (circRNA) is a covalent head-to-tail looped RNA discovered and characterized mostly thanks to the next-generation sequencing techniques in the most recent years (Shen et al., 2015).

Tumor suppression is among the known roles played by circRNAs (Lu et al., 2020). Interestingly, *KLF4* is upregulated by the circRNA circEHMT1 at mRNA and protein levels in breast cancer, consistently with its oncosuppressor role (Lu et al., 2020). Moreover, the circRNA CDR1as affects stemness and proliferation of hepatoblastoma cells *in vitro*. CDR1as interacts with miR-7-5p sponging this miRNA. *KLF4* is a downstream target gene of miR-7-5p and mediates the inhibition of proliferation and stemness (Chen et al., 2020).

## 4 KLF4 in the process of cell plasticity

Cell-fate decisions are concerted processes initiated by TFs that regulate gene expression together with epigenetic modifications (Sardina et al., 2018). Among the most relevant epigenetic modifications there is cytosine methylation in position 5 of the DNA (5mC), which is also relevant in directing the TF binding specificity (Figure 1A) (Sardina et al., 2018). Furthermore, the product of 5mC oxidation (5hmC) recruits chromatin remodelling complexes onto DNA. The B-cell reprogramming into iPSC is accompanied by an almost complete loss of 5mC, with a concomitant increase in 5hmC (Chen et al., 2020). The sustained 5hmC conversion corresponds to an enrichment of specific TFs onto gene regulatory elements (GRE) related to pluripotency and/or genome topology, including KLF4. Furthermore, the demethylation of the core pluripotency TFs (OSKM) leads to their activation.

During pluripotent cell reprogramming, there are some TFs triggering chromatin reconfiguration and TF occupancy. Namely, these are OSKM (Huyghe et al., 2022). Their activity leads to the increase in cellular plasticity and loss of cell specialization in mouse embryonic fibroblasts. This is followed by the transition to induced pluripotent stem cells (iPSC) due to the activation of the pluripotent network (Huyghe et al., 2022).

Transcriptional reprogramming and loss of identity share common characteristics with malignant transformation, both being constrained by oncogenic barriers. The increase of cell plasticity is driven by oncogenes like *KRAS*, whose oncogenic variant displays aspartic acid instead of glycine at position 12 (*KRASG12D*). Simultaneous *p53* deficiency, *c-Myc* expression and *KRASG12D* mutation lead the cell towards plasticity, fostering the early malignant transformation in human cancers (Ischenko et al., 2013; Dost et al., 2020; Marjanovic et al., 2020).

Long non-coding RNAs (lncRNAs) also represent a relevant tool driving cell plasticity by modulating the epigenetic modifications that lead to pluripotency (Du et al., 2021). The lncRNA *Platr10* interacts with *Oct4* promoter and recruits TET1. This latter enzyme is responsible for the site-specific demethylation, a phenomenon known to be the initial step for the initiation of pluripotency. Among the multiple-pluripotency genes modulated through *Platr10* there is also *KLF4* (Du et al., 2021).

Also, pancreatic cancer stem cells (CSCs) result regulated by telomerase activity and telomere length. An interplay between stemness factors and telomere length has been recently reported (Walter et al., 2021). CSCs isolated from patient-derived xenografts (PDXs) present higher telomerase activity and significantly longer telomeres compared to bulk tumor cells. The telomere lengthening depends on the pluripotency/stemness factors OCT3/4, SOX2, NANOG and KLF4, which increase their expression specifically in CSCs. Of note, KLF4 is the one recruited by PARP1 and activating telomerase expression in embryonic stem cells (Hsieh et al., 2017). Interestingly, the upregulation of one of these factors is paralleled by the concomitant upregulation of the others, suggesting a coordinated mechanism of gene expression (Walter et al., 2021). The interaction between the activator of epithelial and pluripotency programmes JMJD3 and KLF4 is the basis for the recruitment of the former on enhancers and promoters of the specific genes (Huang et al., 2020).

The central role of KLF4 in the organization of three-dimensional (3D) enhancers network was elegantly demonstrated in the transition of mouse embryonic fibroblasts to pluripotent stem cells (PSCs) (Di Giammartino et al., 2019). Technologies aimed at defining local or large-scale 3D genomic architecture are represented by targeted or global chromatin conformation capture techniques. These demonstrate differences between somatic and iPSCs together with a strong association with the OSKM TFs, responsible for the somatic cell reprogramming. These reprogramming TFs thus feature an architectural role. *KLF4* depletion, for instance, causes the abrogation of long-distance interactions at specific genomic loci (Wei et al., 2013).

The association of KLF4 with super-enhancers in head and neck squamous cell carcinomas HNSCC is a further evidence of the architectural role that this TF plays in cancer development (Tsompana et al., 2020). The dual oncogenic and tumor suppressor role of KLF4 emerges clearly in the association with chromatin and its structural properties. *KLF4* is the only member of the KLF-family to be upregulated at mRNA and protein levels in human HNSCC epithelial oral cell line SCC25 compared to the primary human gingival epithelial cell line HGEP (Tsompana et al., 2020). ATAC-seq footprints confirmed these data suggesting that *KLF4* is a key regulator in differential active chromatin modifications, activating genes that are relevant for HNSCC (Figure 1E). Further ChIP-seq experiments demonstrated an enrichment in the activatory H3K27ac, H3K4me1, and H3K4me3 histone modifications, bound by KLF4. Super-enhancers, defined by size, TF density, association with lineage-specific TFs and activatory histone modifications, were identified in HNSCC and were bound by KLF4 and deltaNp63 (Figure 1E) (Chapuy et al., 2013; Sethi et al., 2017; Tsompana et al., 2020).

Overall, these evidences confirm the context-dependent role of KLF4 that can act both as a tumor suppressor (as in the case of NHL and CRC) or as a tumor promoter (as in the case of HNSCC).

## 5 Chromatin modifications associated to KLF4 binding

Another role for KLF4, emerged during the past few years, concerns the topological and tridimensional organization of the promoters-enhancers interactions. As known, KLF4 binds to sequences containing methylated CpG (mCpG) (Wan et al., 2017). The trans-activating capabilities of KLF4 are directed towards non-receptor tyrosine kinases like Src tyrosine-kinases which mediate cellular processes such as migration, adhesion, invasion, angiogenesis, proliferation, and differentiation (Oyinlade et al., 2018). In a model of glioblastoma, where *KLF4* was inducible, the issue of the binding to highly-methylated enhancers was addressed. On a total of 3,802 binding fragments, 2,477 had at least one mCpG site (defined by a β value > 0.5) based on whole genome bisulfite sequencing (Oyinlade et al., 2018). The majority of these sites were associated to highly methylated KLF4 binding sites, H3K27ac modifications and located within gene bodies or intergenic enhancers. Another location was upstream of the gene and within the 5’UTR. The B-cell lymphocyte kinase, taken as a model, was upregulated by KLF4 binding to the mCpG at both the promoter region (5’UTR) and enhancer region, located at the 3’UTR (Oyinlade et al., 2018).

A notable example of parentally imprinted genes are the methylation-regulated *IGF2* and *H19* gene clusters (Schagdarsurengin et al., 2017). Within the P0 promoter of *IGF2* there is a differentially methylated region containing a putative KLF4 binding site (DMR0). The hypomethylation of *IGF2-DMR0* is associated to lower *IGF2* expression in PCa tissues compared to benign prostate hyperplasia. KLF4 binds to the hypomethylated *IGF2-DMR0*, leading to reduced *IGF2* mRNA expression (Schagdarsurengin et al., 2017).

## 6 Concluding remarks

The epigenetic regulation of the zinc-finger TF KLF4 plays a relevant role in several types of cancers, as demonstrated by the recent literature. DNA methylation at CpG sites represents a recurrent and widespread epigenetic mechanism for *KLF4* gene silencing in both solid and hematological tumors (Table 1). Histone modifications (either activatory or inhibitory) may influence the chromatin spatial organization, bearing relevant implications for gene silencing.

**TABLE 1:** The main mechanisms of epigenetic regulation affecting *KLF4* expression in tumors. The tumors where the mechanisms have been described are listed and the specific references reported.

Tumor		Epigenetic mechanism involved	References
Hematological tumors	Follicular lymphoma, diffuse large B-cell lymphoma, Burkitt lymphoma (NHL) and cHL. T-ALL	CpG island and global DNA methylation	Guan et al. (2010), Frazzi et al. (2017, 2022), Shen et al. (2017), Bialopiotrowicz et al. (2020), Frazzi et al. (2022)
Solid tumors	Lung adenocarcinoma, hepatocellular carcinoma, CRC, pancreatic cancer, OSCC, ESCC, HNSCC, urothelial cancer, renal cell carcinoma, medulloblastoma, gastric cancer	CpG island and global DNA methylation	Choi et al. (2006), Cho et al. (2007), Xu et al. (2008), Nakahara et al. (2010), Li et al. (2013, 2014, 2015), Wang et al. (2013), Chen et al. (2015), Zhou et al. (2015), Houessinon et al. (2016), Yang and Katz (2016), Xie et al. (2017), Zeng et al. (2018), Li et al. (2021), Cai et al. (2022), Wei et al. (2022)
Hematological tumors	Burkitt lymphoma, T-cell lymphoma	Histone H3K27 trymethylation	Han et al. (2017), Bialopiotrowicz et al. (2020)
	Acute myeloid leukemia	H3K4 demethylation	Faber et al. (2013)
Solid tumors	PCa	Histones H3K9me2 and H3K9me3	Lee et al. (2016)
	Lung cancer	Histone deacetylase inhibition	Yu et al. (2016)
	Glioblastoma, CRC, breast cancer	miRNAs	Miyoshi et al. (2010), Ogawa et al. (2015), Yang et al. (2015)
	Breast cancer	Circular RNA	Lu et al. (2020)

The interactions of DNMTs with the enzymes responsible for histone modifications link several players of the epigenetic scenario, namely, CpG methylation, histone lysine/arginine methylation and, overall, influence the ability of KLF4 to bind and regulate target genes. miRNAs, lncRNAs and circRNAs as well can regulate *KLF4* expression either directly or by modulating histone modifying enzymes.

The last two paragraphs of the present manuscript deal with the two functions played by the TF KLF4 connected to the chromatin conformation/topology. The former is the pivotal role of KLF4 in the reprogramming and generation of iPSC and the latter is represented by the chromatin conformational changes associated to KLF4 binding. In both cases, the CpG methylation emerges as a recurrent epigenetic tool driving further binding and topological events.

Overall, the epigenetic modulation emerges as a pivotal mechanism for regulating *KLF4* functions and therefore represents a potential actionable target.
